# Distinct mechanisms contribute to acquired cisplatin resistance of urothelial carcinoma cells

**DOI:** 10.18632/oncotarget.9321

**Published:** 2016-05-12

**Authors:** Annika Höhn, Katharina Krüger, Margaretha A. Skowron, Stefanie Bormann, Lena Schumacher, Wolfgang A. Schulz, Michèle J. Hoffmann, Günter Niegisch, Gerhard Fritz

**Affiliations:** ^1^ Institute of Toxicology, Medical Faculty, Heinrich Heine University Düsseldorf, 40225, Düsseldorf, Germany; ^2^ Department of Urology, Medical Faculty, Heinrich Heine University Düsseldorf, 40225, Düsseldorf, Germany

**Keywords:** cisplatin, urothelial carcinoma, DNA damage response, DNA repair, cisplatin resistance

## Abstract

Cisplatin (CisPt) is frequently used in the therapy of urothelial carcinoma (UC). Its therapeutic efficacy is limited by inherent or acquired drug resistance. Here, we comparatively investigated the CisPt-induced response of two different parental urothelial carcinoma cell lines (RT-112, J-82) with that of respective drug resistant variants (RT-112^R^, J-82^R^) obtained upon month-long CisPt selection. Parental RT-112 cells were ~2.5 fold more resistant to CisPt than J-82 cells and showed a different expression pattern of CisPt-related resistance factors. CisPt resistant RT-112^R^ and J-82^R^ variants revealed a 2–3-fold increased CisPt resistance as compared to their corresponding parental counterparts. Acquired CisPt resistance was accompanied by morphological alterations resembling epithelial mesenchymal transition (EMT). RT-112^R^ cells revealed lower apoptotic frequency and more pronounced G2/M arrest following CisPt exposure than RT-112 cells, whereas no differences in death induction were observed between J-82 and J-82^R^ cells. CisPt resistant J-82^R^ cells however were characterized by a reduced formation of CisPt-induced DNA damage and related DNA damage response (DDR) as compared to J-82 cells. Such difference was not observed between RT-112^R^ and RT-112 cells. J-82^R^ cells showed an enhanced sensitivity to pharmacological inhibition of checkpoint kinase 1 (Chk1) and, moreover, could be re-sensitized to CisPt upon Chk1 inhibition. Based on the data we suggest that mechanisms of acquired CisPt resistance of individual UC cells are substantially different, with apoptosis- and DDR-related mechanisms being of particular relevance. Moreover, the findings indicate that targeting of Chk1 might be useful to overcome acquired CisPt resistance of certain subtypes of UC.

## INTRODUCTION

Bladder cancer is a frequent type of cancer world-wide. In most countries, the majority of bladder cancers are urothelial carcinoma (UC) [1]. Cisplatin (CisPt)-based therapeutic regimen are commonly used both in the perioperative (neoadjuvant, adjuvant) setting for muscle-invasive UC as well as in the palliative setting for recurrent or metastatic UC [2]. CisPt enters cells by passive diffusion as well as by help of transporters [3, 4]. Upon replacement of its chloride ligands by water, DNA adducts are formed by S_N_2 mechanism (nucleophilic substitution) [5]. The vast majority (60–80%) of DNA adducts generated by CisPt are DNA intrastrand crosslinks (GpG and ApG). Only 1–2% of the DNA crosslinks formed by CisPt are DNA interstrand crosslinks [6, 7]. Platinum-induced DNA crosslinks cause a substantial distortion of the DNA double helix, resulting in transcription and replication blockage [8, 9]. In consequence of stalled replication forks, DNA double-strand breaks (DSBs) can arise as secondary lesions [10]. DSBs are potent triggers of cell death [11] and can be repaired by DNA double-strand break repair (homologous recombination (HR) or non-homologous end joining (NHEJ)). The removal of CisPt-induced DNA crosslinks involves nucleotide excision repair (NER), including transcription-coupled NER (TC-NER) [9]. The relevance of NER mechanisms for the tumor cell response to CisPt is highlighted by the fact that the expression of the NER factor ERCC1 predicts the therapeutic efficacy of CisPt in lung tumors [12, 13] and also seems to be of relevance for UC [14]. The efficacy of platinum-based therapy is limited by intrinsic or acquired drug resistance [15]. Factors that contribute to CisPt resistance are manifold and are poorly characterized for UC [16, 17]. Recently, mechanisms affecting resistance to CisPt have been classified according to their site of action as pre-, on-, post- and off-target [17], with drug transport, DNA repair, apoptosis and signal transduction at membranes, respectively, being representatives of these mechanisms.

Upon induction of DNA damage a highly complex cellular stress response program, known as the DNA damage response (DDR), is activated. It tightly controls cell cycle progression by activation of cell cycle checkpoints and fine-tunes mechanisms of DNA repair and cell death [18, 19]. Activation of the DDR is considered as an inducible barrier against early tumorigenesis [20, 21] and, moreover, to precede genomic instability in bladder cancer [22]. In case of error prone repair of DSBs, genomic instability of bladder carcinomas is favoured [23]. DSBs as well as replication- and transcription-blocking DNA lesions are particular efficient activators of the DDR. The PI3-like kinases ATM and ATR play key roles in the regulation of the DDR [24, 25]. These kinases phosphorylate numerous substrates, among others checkpoint kinases (e.g. Chk1, Chk2) and p53, which eventually affect survival or death of the damaged cell. The relevance of DDR mechanisms for the CisPt sensitivity of UC cells and, most importantly, for CisPt resistant variants, is largely unknown.

Urothelial cancer cells segregate into epithelial and mesenchymal subsets [26]. Therefore, we included both RT-112 and J-82 cells, which are described as UC cells of mainly epithelial- and mesenchymal-like phenotype, respectively [27, 28], in our study. To select CisPt resistant variants we took into account that the therapeutic regimen commonly used in CisPt-based anticancer therapy comprises repetitive treatment cycles, where CisPt is administered by infusion, intermitted by treatment free periods. Therefore, parental RT-112 and J-82 UC cells were selected for resistance by multiple pulse-treatments with CisPt followed by extended recovery periods. The aim of the study was to comparatively analyze CisPt resistant UC cell variants (RT-112^R^ and J-82^R^) with their respective parental cell types regarding (i) DDR capacity, (ii) the expression of putative CisPt resistance factors as suggested by Galluzzi et al. [17] and (iii) the response to a subset of pharmacological modifiers of the DDR and DNA repair, including inhibitors of checkpoint kinases, which are promising novel anticancer compounds acting by selectively increasing replicative stress and cell death in malignant cells [29]. In doing so, we aimed to identify mechanisms that are of relevance for acquired CisPt resistance of UC cells and, moreover, to figure out therapeutic options to overcome their CisPt resistant phenotype.

## RESULTS AND DISCUSSION

### Characterization of the CisPt response of the urothelial carcinoma cells RT-112 and J-82

**Figure 1:**
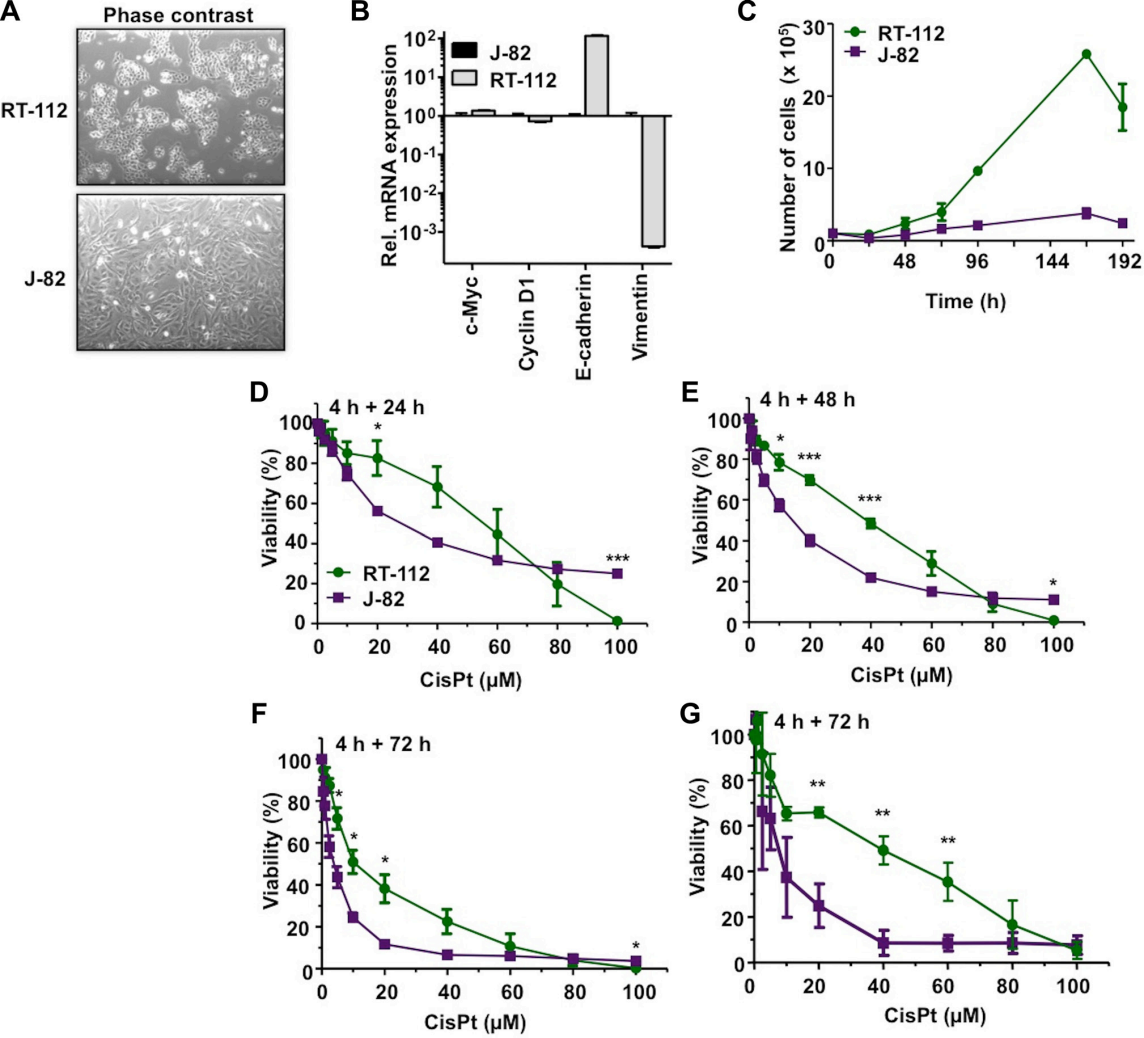
Differential CisPt sensitivity of urothelial carcinoma cells RT-112 and J-82 (**A**) Different morphology of RT-112 and J-82 cells. (**B**) Quantitative real-time PCR-based mRNA expression analysis (qRT-PCR) of epithelial (*E-cadherin*) and mesenchymal (*vimentin*) markers in J-82 and RT-112 cells. For control, mRNA expression of *c-Myc* and *CyclinD1* was analyzed as well. Relative mRNA expression in J-82 cells was set to 1.0. Data shown are the mean ± SD from one experiment performed in triplicate. (**C**) Cell growth of RT-112 and J-82 cells was monitored by determining the number of cells over a total period of 8 days. Data shown are the mean ± SD from two to three independent experiments each performed in duplicate. (**D**–**G**) Logarithmically growing cells were pulse-treated with different concentrations of cisplatin (CisPt) for 4 h. After post-incubation period of 24 h (D), 48 h (E) or 72 h (F, G) in the absence of CisPt, cell viability was analyzed using the Alamar blue assay (D–F) or the Neutral red assay (G). Data shown are the mean ± SD from three independent experiments, each performed in triplicate. *statistical significance of RT-112 cells vs. J-82 cells. ***p ≤ 0.001; **p ≤ 0.01; *p ≤ 0.05.

**Figure 2:**
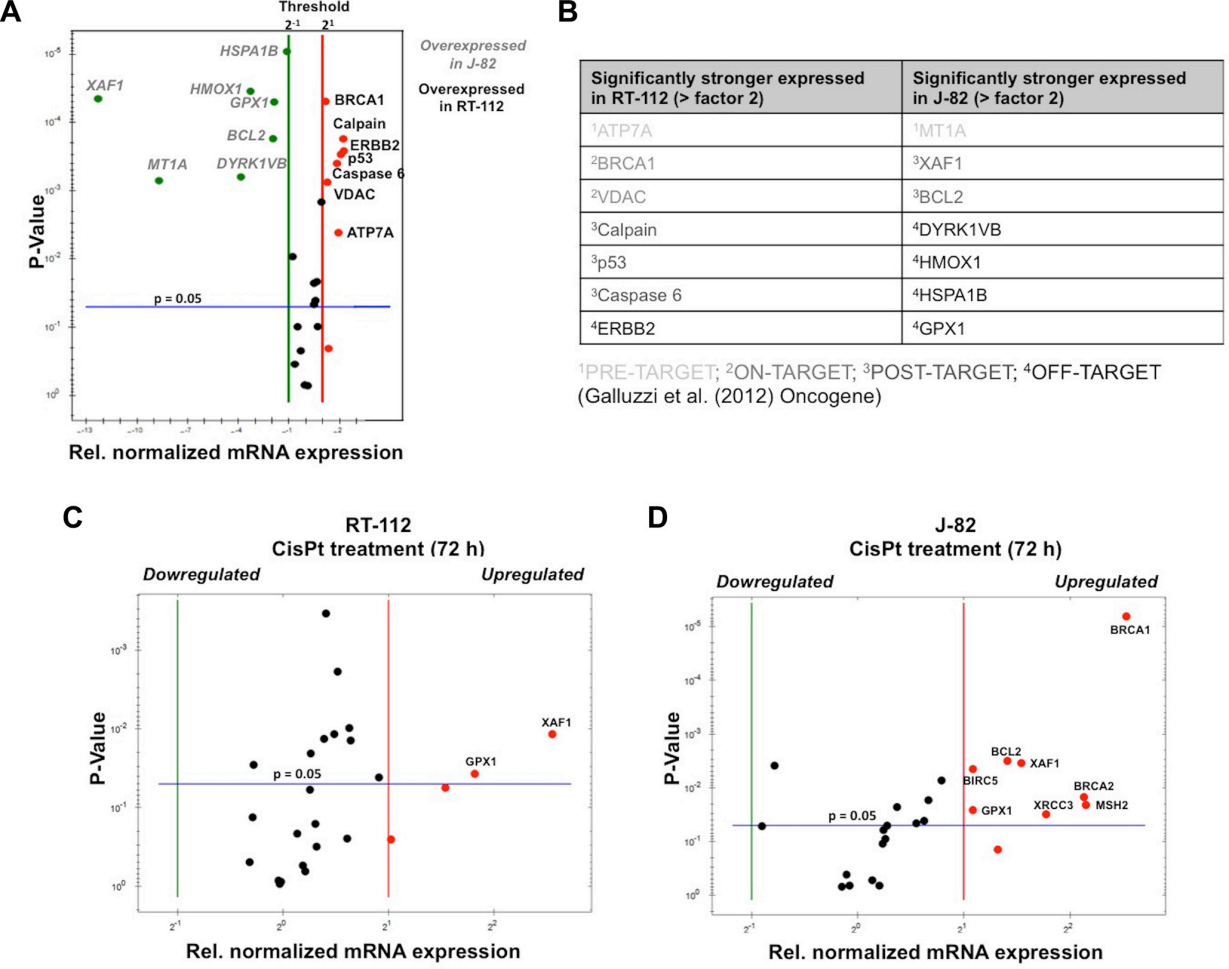
Basal and CisPt-induced mRNA expression of CisPt-related susceptibility factors in UC cells (**A**) Basal mRNA expression of CisPt susceptibility factors [17] was analyzed by qRT-PCR analysis. The mean values shown are based on two independent experiments each performed in triplicate. Only differences in mRNA expression of ≤ 0.5 or ≥ 2.0 were considered as biologically relevant. (**B**) Variations in basal mRNA expression of factors related to CisPt resistance between RT-112 and J-82 cells are classified into mechanisms of pre-, on-, post- and off-target resistance according to Galluzzi et al. [17]. (**C**, **D**) mRNA expression of CisPt susceptibility factors was analyzed by qRT-PCR analysis 72 h after treatment with the IC_50_ of CisPt (according to Figure 1F). The mean values shown are based on a representative experiment performed in triplicate. Only differences in mRNA expression of ≤ 0.5 or ≥ 2.0 were considered as biologically relevant.

In light of the fact that urothelial cancer cells can segregate into epithelial and mesenchymal subsets [26], RT-112 und J-82 cells, which are representative of epithelial- and mesenchymal-like UC cells, respectively [27, 28], were used in the present study. RT-112 cells differ from J-82 regarding morphology (Figure 1A) and a higher mRNA expression of the epithelial marker *E-cadherin* as well as a lower expression of the mesenchymal marker *vimentin* (Figure 1B) as expected. Proliferation rate was higher in RT-112 as compared to J-82 cells (Figure 1C). Analyzing the influence of CisPt on cell viability 24–72 h after CisPt pulse-treatment, we observed that RT-112 cells are 2–3-fold more resistant to moderate doses of CisPt than J-82 cells (Figure 1D–1F). This is reflected by IC_50_/IC_80_ values of 10.7 μM / 44.3 μM and 3.9 μM / 13.5 μM for RT-112 and J-82, respectively, as determined after a post-incubation period of 72 h by the Alamar blue assay (Figure 1F). This difference in drug sensitivity is not detectable anymore at very high CisPt doses of ≥ 80 μM (Figure 1D–1G). Measuring cell viability via an alternative method, i.e. the Neutral red assay, similar results were obtained (Figure 1G). Based on a recent report of Galluzzi et al. [17], who has classified putative CisPt resistance factors of tumor cells, we assembled a 96 well-based quantitative real-time (qRT) PCR array to comparatively analyze the mRNA expression of these factors in RT-112 and J-82 cells. The results of this analysis revealed large cell type-specific differences in the basal mRNA expression of both pre-, on-, post- as well as off-target factors [17]. In more detail, we observed a significantly stronger mRNA expression of *ATP7A, BRCA1, VDAC, Calpain, p53, Caspase 6* and *ERBB2* in RT-112 cells as compared to J-82 cells. By contrast, J-82 cells revealed an enhanced expression of *MT1A, XAF1, BCL2, DYRK1VB, HMOX1, GPX1* and *HSPA1B* as compared to RT-112 cells (Figure 2A, 2B). Analysing gene expression 72 h after treatment with the IC_50_ of CisPt, we found upregulation of *GPX1* and *XAF1* concommitantly in both RT-112 and J-82 cells (Figure 2C, 2D). Notably, J-82 cells responded to CisPt treatment with the upregulation of various DNA repair-related factors (i.e. *BRCA1, BRCA2, MSH2, XRCC3*) (Figure 2D). This response was not found in RT-112 cells (Figure 2C). Taken together, the data show that both basal and CisPt-stimulated mRNA expression of factors affecting CisPt sensitivity [17] considerably vary between the two examined UC cell lines, indicating that the basal defence capacity of epithelial- and mesenchymal-like UC cells against CisPt-induced injury might be different. This hypothesis needs future confirmation by analyzing the CisPt response of additional UC cell lines of epithelial or mesenchymal origin both *in vitro* and *in vivo*.

### Selection of CisPt resistant UC cell variants

**Figure 3:**
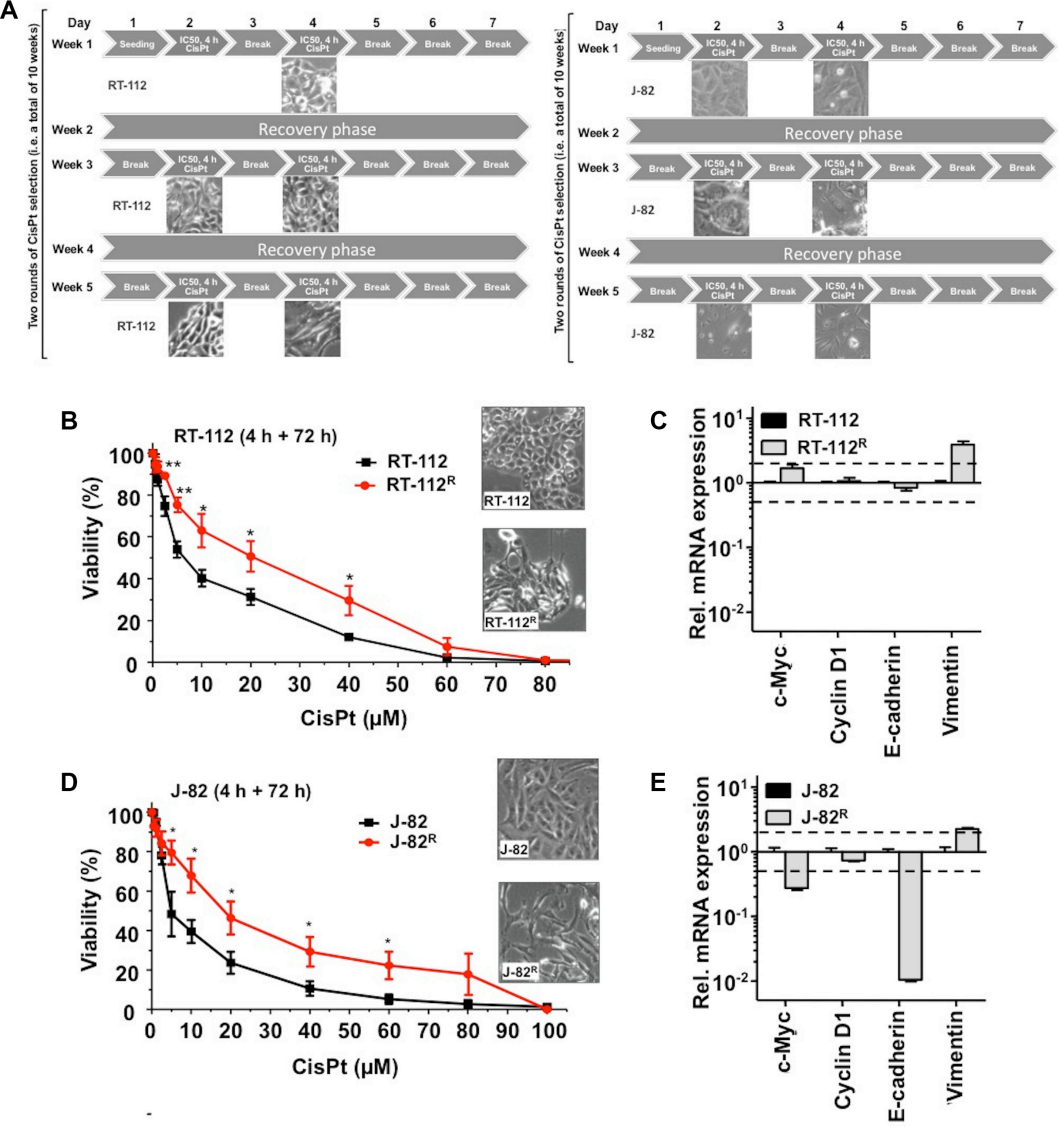
CisPt resistant UC cell variants obtained by long-term selection with CisPt display an intensified mesenchymal phenotype (**A**) Schematic representation of the long-term CisPt selection scheme applied to RT-112 and J-82 cells. Cells were pulse-treated with the corresponding IC_50_ of CisPt (according to Figure 1F) twice a week, followed by a recovery period of one week. This selection scheme was performed over a total time period of 10 weeks (shown are only the first 5 weeks). (**B**, **D**) Cell viability of parental RT-112 and CisPt selected RT-112^R^ cells (B) or of parental J-82 and CisPt selected J-82^R^ cells (D) was measured 72 h after a 4 h pulse-treatment with different concentrations of CisPt using the Alamar blue assay. Data shown are the mean ± SD from three independent experiments each performed in quadruplicate. The microscopic pictures illustrate the cell morphology of parental and CisPt resistant cells. *statistical significance of parental cells vs. CisPt resistant cells. **p ≤ 0.01; *p ≤ 0.05. (**C**, **E**) Alterations in the mRNA expression of marker genes of epithelial-mesenchymal transition (EMT) in RT-112 versus RT-112^R^ cells (C) or J-82 versus J-82^R^ cells (E). The qRT-PCR based data shown are the mean ± SD from triplicate determinations.*E-cadherin* is a representative marker of epithelial cells while *vimentin* is a prototypical marker of mesenchymal cells. For control, mRNA expression of *c-Myc* and *CyclinD1* were also determined.

**Figure 4:**
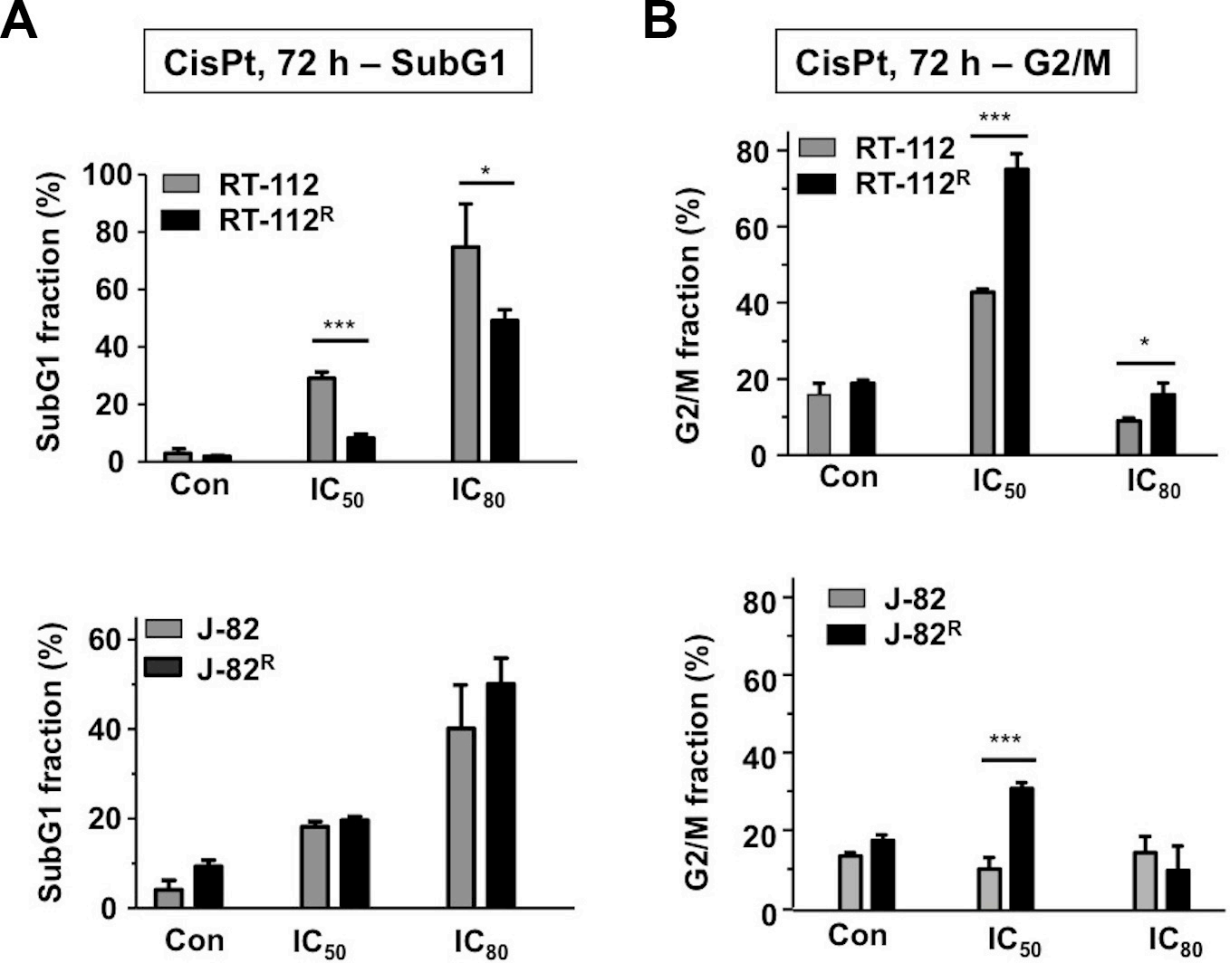
Effects of CisPt on cell cycle distribution of parental and CisPt resistant UC cells (**A**, **B**) Parental (RT-112, J-82) and CisPt resistant (RT-112^R^, J-82^R^) UC cells were treated with the IC_50_ or IC_80_ of CisPt (according to Figure 1F). After incubation period of 72 h, subG1 fraction (A) and cells present in G2/M phase of the cell cycle (B) were determined by flow cytometry-based analyses. Data shown are the mean ± SD from three independent experiments each performed in duplicate. *statistical significance of parental cells vs. CisPt resistant cells. ***p ≤ 0.001; *p ≤ 0.05.

In order to elucidate which mechanisms contribute to acquired CisPt resistance of UC cells and having in mind the therapeutic regimen used in the clinic, RT-112 and J-82 cells were repeatedly pulse-treated twice a week (for each 4 h) with the corresponding IC_50_ of CisPt, followed by a recovery period of one week (Figure 3A). After a total selection time of 10 weeks, CisPt resistant RT-112^R^ und J-82^R^ cells were obtained (Figure 3B–3D). Measuring cell viability by the Alamar blue assay, the resistant variants revealed an about 3-fold increase in the IC_50_ as compared to the corresponding parental cells (Figure 3B–3D). Similar results were obtained using the Neutral red assay (data not shown). Gain of CisPt resistance was accompanied by morphological alterations, in particular cell enlargement and distinct cell protrusions (Figure 3B–3D). Both RT-112^R^ and J-82^R^ cells showed an increased mRNA expression of the intermediate filament vimentin (Figure 3C–3E) as compared to their respective parental cells. As vimentin expression represents a prototypical marker of mesenchymal cells, we hypothesize that the development of an EMT-like phenotype is favoured in epithelial-like RT-112 cells and is further promoted in J-82 cells during the selection of CisPt resistant UC cell variants. A coherence between EMT and acquired drug resistance was reported by others [26, 30–32]. Flow cytometry-based analyses performed 72 h after CisPt treatment showed a reduction of apoptotic cell death in RT-112^R^ cells as compared to RT-112 (Figure 4A). This effect was only observed in RT-112^R^ cells (Figure 4A, upper panel) but not in J-82^R^ cells (Figure 4A, lower panel). Both RT-112^R^ and J-82^R^ cells were characterized by a more pronounced activation of G2/M checkpoint mechanisms as compared to their corresponding parental counterparts (Figure 4B). The data show that the mechanisms of acquired CisPt resistance differ between individual UC cell lines with protection from CisPt-induced apoptotic mechanisms and alterations in checkpoint control mechanisms being involved.

### Induction of DNA damage and activation of the DNA damage response (DDR) in parental and CisPt resistant UC cell variants

**Figure 5:**
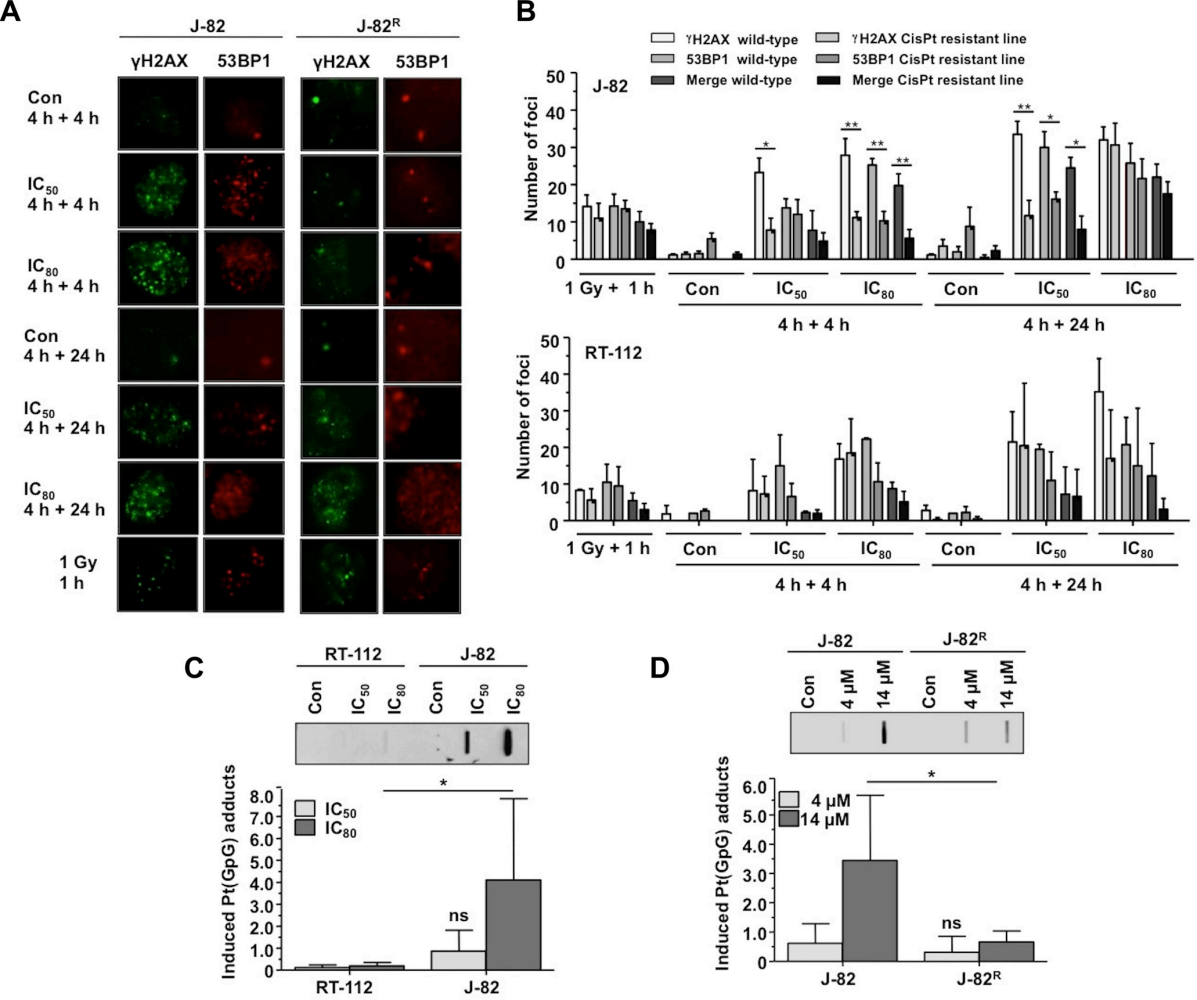
Formation and repair of DNA damage in parental UC cells and CisPt resistant UC variants (**A**, **B**) Parental (RT-112, J-82) and CisPt resistant (RT-112^R^, J-82^R^) UC cells were pulse-treated for 4 h with the IC_50_ or IC_80_ of CisPt (according to Figure 1F) for 4 h. After a post-incubation period of 4 h or 24 h in the absence of CisPt, the formation of nuclear γH2AX and 53BP1 foci was analyzed by immunocytochemistry. Data shown are the mean ± SD from three independent experiments with each ≥ 50 nuclei being analyzed per experiment. (A) representative microscopic pictures from J-82 and J-82^R^ cells. (B) histograms with quantitative data from J-82/J-82^R^ cells (upper panel) and RT-112/RT-112^R^ cells (lower panel). (**C**) RT-112 and J-82 cells were pulse-treated for 4 h with the IC_50_ or IC_80_ of CisPt (according to Figure 1F). The level of Pt-(GpG) intrastrand crosslinks was determined by Southwestern analyzes using an anti-Pt-(GpG)-specific antibody. Autoradiographies were analyzed densitometrically and the signal intensities of the untreated controls were subtracted. The upper part of the figure shows the result of a representative experiment. In the lower part, mean values ± SD from two independent experiments each performed in triplicate are shown. Con, untreated control. *statistical significance of RT-112 versus J-82. ns, not significant. (**D**) Parental (J-82) and CisPt resistant cells (J-82^R^) were pulse-treated for 4 h with the indicated CisPt dose. The level of Pt-(GpG) intrastrand crosslinks was determined by Southwestern analyzes using an anti-Pt-(GpG)-specific antibody. Autoradiographies were analyzed densitometrically and the signal intensities of the untreated controls were subtracted. The upper part of the figure shows the result of a representative experiment. In the lower part, mean values ± SD from two independent experiments each performed in triplicate are shown. Con, untreated control. *statistical significance of J-82 versus J-82^R^ (*p ≤ 0.05). ns, not significant.

In order to measure the induction of DNA damage following CisPt treatment, ATM/ATR-catalyzed S139 phosphorylation of histone H2AX and the recruitment of 53BP1 to sites of damage were monitored by immunocytochemistry (Figure 5A–5B). Moreover, the level of CisPt-induced DNA intrastrand crosslinks was monitored by Southwestern analysis (Figure 5C–5D). The formation of nuclear γH2AX foci and 53BP1 foci is part of the DNA damage response (DDR) and is believed to reflect predominantly the formation of DNA double-strand breaks (DSBs) [19]. Following CisPt treatment, DSBs are believed to be mainly generated as secondary lesions from primary DNA platinum-adducts that stall replication forks [10]. As observed 4 h and 24 h after CisPt pulse-treatment for 4 h, we found a significant reduction in the number of DSBs in J-82^R^ cells, but not in RT-112^R^ cells (Figure 5A–5B). This finding indicates that CisPt resistance of J-82^R^ cells, but not of RT-112^R^ cells, might result from a reduced formation of highly cytotoxic DSBs and/or attenuated DDR following CisPt treatment. Bearing in mind that CisPt-induced DSBs mainly originate from primary Pt-(GpG) DNA adducts, we next monitored the formation of Pt-(GpG) intrastrand crosslinks by Southwestern blot analyses. The data show that DNA intrastrand crosslink formation was significantly lower in the J-82^R^ subline as compared to J-82 parental cells (Figure 5D). Based on these observations we suggest that acquired CisPt resistance of J-82 cells involves a reduced formation of primary (i.e. Pt-(GpG) adducts) and secondary (i.e. DSBs) DNA damage following CisPt treatment. Mechanistically, it is feasible that pre-target resistance mechanisms such as transport or detoxification mechanisms take part [17]. In this context it is noteworthy that the level of CisPt-induced Pt-(GpG) DNA intrastrand crosslinks is higher in parental J-82 cells as compared to RT-112 cells (Figure 5C) if the corresponding IC_50_ and IC_80_ were used. This finding indicates that the level of Pt-(GpG) intrastrand crosslinks does not necessarily predicts the level of cytotoxicity.

**Figure 6:**
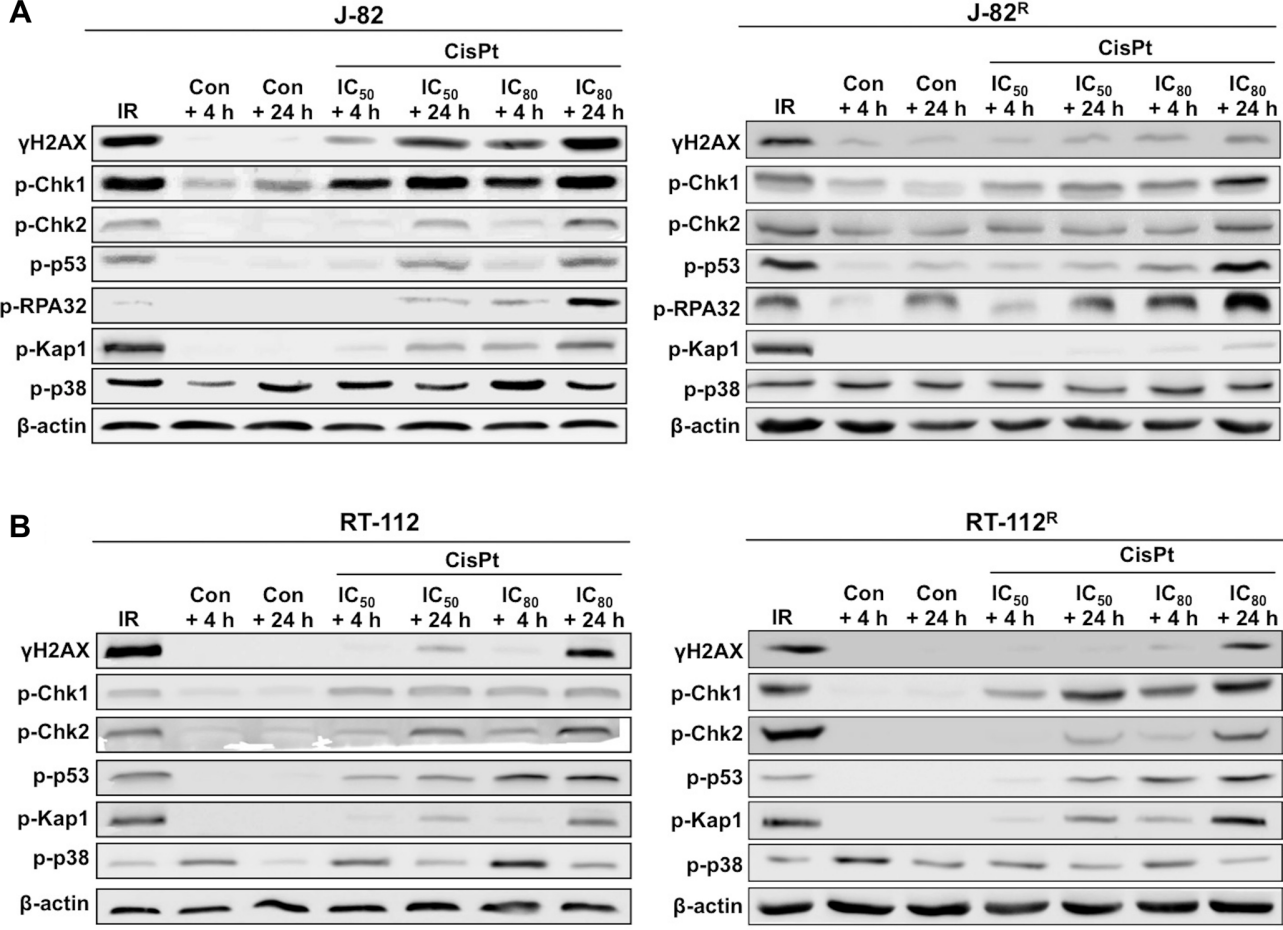
Comparative analyzes of CisPt-induced mechanisms of the DNA damage response (DDR) in parental and CisPt resistant cells Parental (J-82 (**A**) and RT-112 (**B**)) and CisPt resistant (J-82^R^ (A) and RT-112^R^ (B)) cells were treated with the IC_50_ or IC_80_ of CisPt (according to Figure 1F) for 4 h. After post-incubation periods of 4 h or 24 h cells were harvested for Western blot analyses using phospho-specific antibodies as indicated. For control, cells were irradiated with 10 Gy (IR) and analysis was performed 1 h later. Data shown are representative of two independent experiments. Expression of β-actin was determined as protein loading control.

In a next step we comparatively analyzed the DDR of UC parental cells and corresponding CisPt resistant variants following CisPt treatment by Western blot analysis. The data obtained uncover large variations in the activation of DDR mechanisms already in J-82 versus RT-112 parental cells, as reflected on the levels of γH2AX, p-Chk1, p-p53 and p-Kap1 (Figure 6A–6B). In general, J-82 revealed a more profound activation of the DDR than RT-112 cells. This is likely related to the higher level of Pt-(GpG) adducts in J-82 cells (see Figure 5C), resulting in stronger activation of DDR mechanisms. Comparative analyses of J-82 cells versus CisPt resistant J-82^R^ cells showed lower phosphorylation levels of H2AX, Chk1, p53 and Kap1 in the CisPt resistant variants (Figure 6A). Again, this is in line with the observed decrease in Pt-(GpG) DNA adducts and DSB formation described in J-82^R^ as compared to J-82 cells (see Figure 5). RT-112^R^ cells revealed a specifically increased phosphorylation of Chk1 as compared to RT-112 parental cells (Figure 6B). This is indicative of a selectively increased potency of RT-112^R^ cells to activate checkpoint control mechanisms that might contribute to protection from CisPt induced apoptotic mechanisms (see Figure 4A).

### Expression of CisPt susceptibility factors in CisPt resistant UC cells

**Figure 7:**
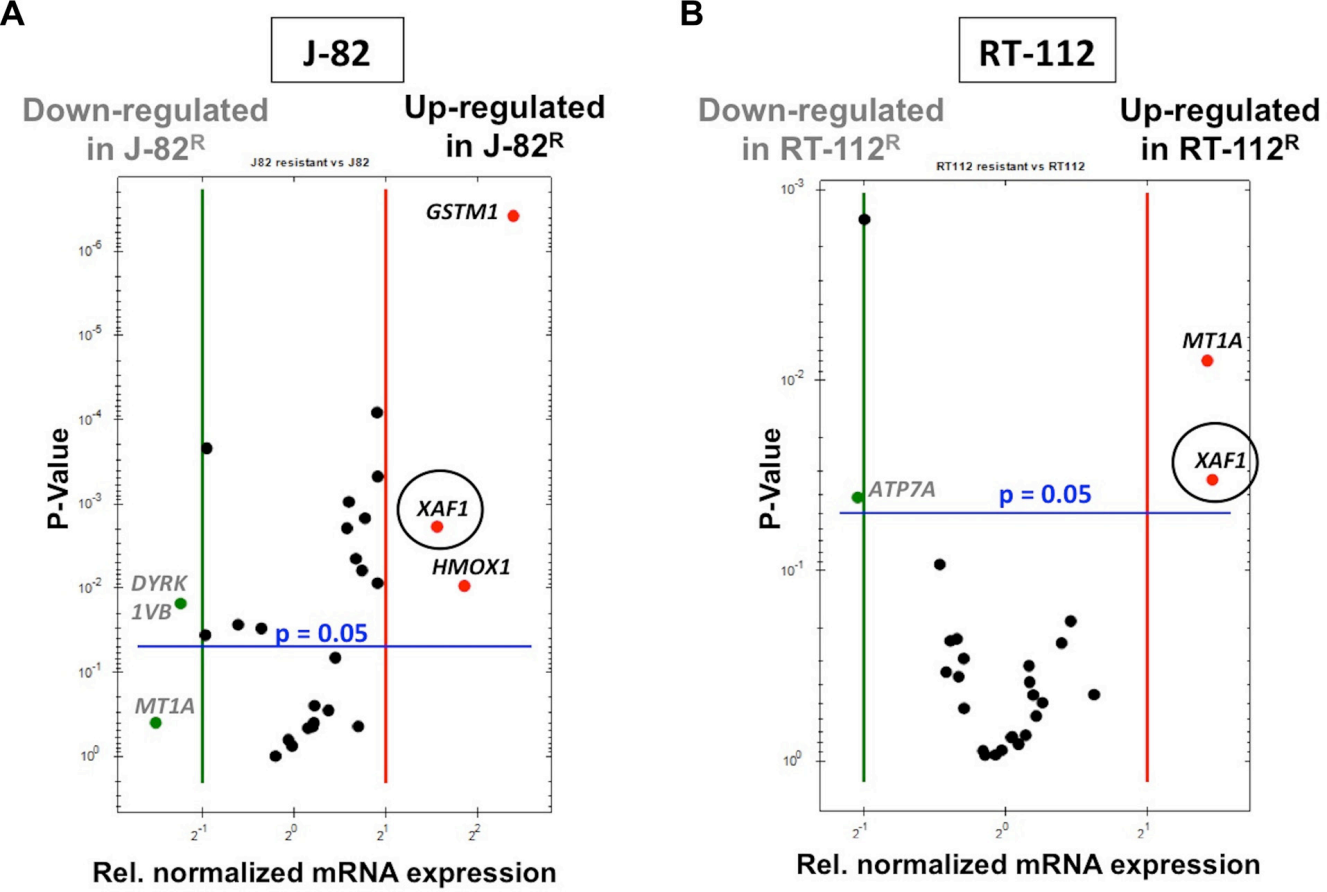
Alterations in gene expression that go along with acquired CisPt resistance of epithelial- and mesenchymal-like UC cells Alterations in the mRNA expression of selected subset of CisPt-related susceptibility factors [17] was analyzed in drug resistant J-82^R^ (**A**) and RT-112^R^ cells (**B**) as compared to the corresponding parental cells by qRT-PCR. Relative mRNA expression in parental J-82 cells was set to 1.0. Only alterations in gene expression of ≤ 0.5 or ≥ 2.0 between wild-type (J-82 and RT-112) and CisPt resistant variants (J-82^R^ and RT-112^R^) were considered as biologically relevant. Shown are the genes that are either up- or downregulated in CisPt resistant cells as compared to the parental cells.

Next, we analyzed the mRNA expression of CisPt-related susceptibility factors reported by Galluzzi et al. [17] in RT-112^R^ and J-82^R^ cells as compared to the corresponding parental cells. Regarding RT-112^R^ cells, we found a significant increase in the mRNA expression of metallothionein (*MT1A*) and the XIAP-associated factor 1 (*XAF1*) as compared to RT-112 cells (Figure 7B). In J-82^R^ cells we observed an elevated mRNA expression of the antioxidative factors heme oxygenase 1 (*HMOX1*) and glutathione S-transerase M1 (*GSTM1*) as well as of *XAF1* as compared to the corresponding controls (Figure 7A). Hence, the two types of CisPt resistant UC cell variants were characterized by an increased mRNA expression of *XAF1*. In this context we would like to note that selection of CisPt resistant J-82 and RT-112 cells by a selection protocol using continuous treatment with increasing CisPt doses over a time period of 4 month also resulted in increased level of *XAF1* mRNA in CisPt resistant J-82 cells but not in RT-112 cells (Supplementary Figure S1). The finding of upregulated *XAF1* mRNA expression in drug resistant UC cell variants was unexpected considering that XAF1 is known to inhibit the anti-apoptotic factor XIAP, and hence is anticipated to promote cell death [33]. Correspondingly, high XAF1 level was suggested as predictive marker in pancreatic cancer associated with better overall survival [34]. Therefore, it appears possible that its increased mRNA expression in J-82^R^ cells accidentially correlates with CisPt resistance but is not causative for acquired CisPt resistance of UC cells. Alternatively, XAF1 might have a so far not yet decribed pro-survival function in CisPt resistant UC cells. In this context it is noteworthy that a cell cycle regulatory function has been suggested for XAF1 in gastrointestinal cancer, which rests on its interaction with Chk1 [35]. Interestingly enough induction of *XAF1* mRNA expression was also observed in both J-82 and RT-112 parental cells 72 h after CisPt addition (see Figure 2C–2D). So, forthcoming studies are clearly required to dissect the role of XAF1 in the response of UC cells to CisPt. In addition, the data indicate that the improvement of anti-oxidative capacity, as reflected by the upregulation of *HMOX1* and *GSTM1*, and increased expression of metallothionein *MT1A* might be of particular relevance for acquired CisPt resistance of some subtypes of UC. Bearing in mind that oxidative stress contributes to the cytotoxicity of CisPt [36, 37], upregulation of anti-oxidative mechanisms might be a meaningful cytoprotective strategy of UC cells, as is the upregulation of metallothioneins [38]. Noteworthy, upregulation of the mRNA expression of DNA repair factors (i.e. *BRCA1, BRCA2, ERCC1, MLH1, MSH2, XRCC3*), which are involved in the repair of CisPt-induced DNA damage, was not observed in the CisPt resistant variants.

### J-82^R^ cells show enhanced sensitivity to a Chk1 inhibitor

In search of pharmacological approaches to overcome acquired CisPt resistance of J-82^R^ cells, we examined their sensitivity to a selected subset of pharmacological inhibitors. Unfortunately, these analyses could not be performed with RT-112^R^ cells because their CisPt resistant phenotype turned out as not stable and got lost upon freezing. For these analyses inhibitors of the DDR-related kinases ATM/ATR (VE-822) as well as of checkpoint (Chk) kinases (AZD-7762 (Chk1 and Chk2 inhibitor) and LY2603618 (Chk1-specific inhibitor)) and Wee1 kinase (MK-1775) were included. Noteworthy, targeting of ATR/Chk1-regulated replicative stress responses of tumor cells has recently been suggested as a novel therapeutic strategy [29]. As additional candidate inhibitors we analyzed the impact of the cyclin-dependent kinase (CDK) inhibitor roscovitine, the multikinase inhibitor sorafenib, which is frequently used as anticancer drug in the clinic, as well as of inhibitors of the DNA repair proteins RAD51 (RI-1) and PARP-1 (olaparib) on the viability of parental J-82 versus resistant J-82^R^ cells. As a further candidate inhibitor we employed lovastatin, because statins have been shown to exhibit anticancer activity in various preclinical model systems [39] and are discussed to overcome acquired drug resistance to doxorubicin in neuroblastoma cells [40].

**Figure 8:**
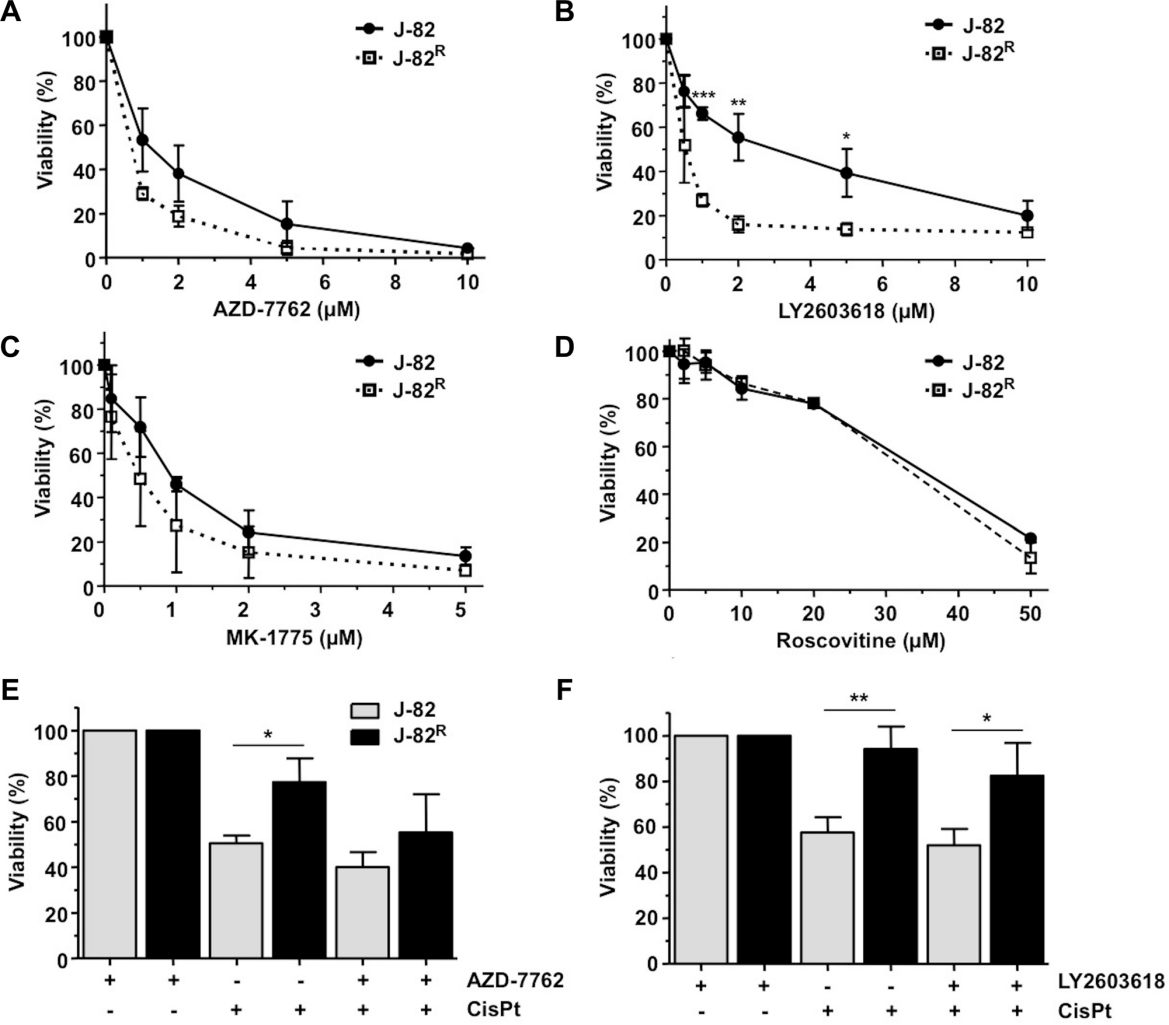
Influence of selected protein kinase inhibitors on the viability of CisPt resistant J-82^R^ cells (**A**–**D**) J-82 cells and CisPt resistant cells (J-82^R^) were treated with different concentrations of the pan Chk inhibitor AZD-7762 (A), the Chk1-specific inhibitor LY2603618 (B), the Wee1 kinase inhibitor MK-1775 (C) or the cyclin dependent kinase inhibitor roscovitine (D). After an incubation period of 72 h in the presence of the corresponding inhibitor, viability was analyzed using the Alamar blue assay. Data shown are the mean ± SD from three independent experiments, each performed in quadruplicate. *statistical significance of parental cells vs. CisPt resistant cells. ***p ≤ 0.001; **p ≤ 0.01; *p ≤ 0.05. (**E**–**F**) J-82 parental and CisPt resistant cells (J-82^R^) were pre-treated with 0.4 μM of the pan Chk inhibitor AZD-7762 (E) or the Chk1-specific inhibitor LY2603618 (F) for 1 h followed by the addition of CisPt (2 μM). After further incubation period of 72 h in the absence of the Chk inhibitor, cell viability was analyzed using the Alamar blue assay. Relative viability in the corresponding inhibitor-only treated controls was set to 100%. Data shown are the mean ± SD from three independent experiments, each performed in quadruplicate. *statistical significance of parental cells vs. CisPt resistant cells. **p ≤ 0.01; *p ≤ 0.05.

J-82^R^ cells turned out to be slightly more sensitive to treatment with the pan Chk inhibitor AZD-7762 (Figure 8A) and showed a significantly enhanced sensitivity to the Chk1-specific inhibitor LY2603618 as compared to parental cells (Figure 8B). The J-82^R^ cells also revealed a tendentially enhanced sensitivity to the Wee1 kinase inhibitor MK-1775 (Figure 8C) but not to the CDK inhibitor roscovitine (Figure 8D). The pronounced loss of cell viability of J-82^R^ cells following Chk1 inhibition seems to be specific as it was not observed upon inhibition of ATM/ATR kinase or the DNA repair factors RAD51 and PARP-1 (Table 1). Pre-treatment of J-82^R^ cells with low non-toxic concentration of Chk inhibitors increased their sensitivity to CisPt (Figure 8E–8F), indicating that targeting of Chk might be particular useful to overcome acquired CisPt resistance of some subtypes of UC cells. Whether targeting of Chk is equally effective in epithelial and mesenchymal-like UC cells remains to be elucidated in forthcoming studies. Noteworthy, Chk inhibition was reported to overcome CisPt resistance of head and neck cancer cells [41] as well as of clear cell carcinoma of the ovary [42] *in vitro*, supporting the hypothesis that targeting of Chk might be a useful approach to deal with acquired CisPt resistance of different types of tumor cells. Taken together, our data support the current view that increasing replicative stress in tumor cells might be a promising therapeutic strategy also in UC [29]. In fact, the antitumor potency of Chk1 inhibitors is currently investigated in clinical trials. Whereas AZD-7762 revealed inacceptable cardiotoxicity [43], SCH-900776 seems to be better tolerated in humans [44].

**Table 1:** Influence of selected pharmacological modulators of the DNA damage response (DDR) and of DNA repair factors on the viability of parental and CisPt resistant J-82 cells

Inhibitor	Dose	Cell line	
		J-82	J-82^R^
AZD-7762	IC_50_	1.2 μM	0.7 μM
	IC_80_	4.4 μM	1.8 μM
LY2603618	IC_50_	2.82 μM	0.54 μM
	IC_80_	9.85 μM	1.63 μM
MK-1775	IC_50_	0.92 μM	0.47 μM
	IC_80_	3.1 μM	1.7 μM
VE-822	IC_50_	~ 10 μM	> 10 μM
Roscovitine	IC_50_	25 μM	35 μM
Sorafenib	IC_50_	9 μM	> 10 μM
RI-1	IC_50_	150 μM	140 μM
Olaparib	IC_50_	375 μM	347 μM
Lovastatin	IC_50_	26 μM	> 30 μM

J-82 cells and the CisPt resistant subline (J-82^R^) were treated with different concentrations of the pan Chk inhibitor AZD-7762, the Chkl-specific inhibitor LY2603618, the Wee1 kinase inhibitor MK-1775, the ATM/ATR inhibitor VE-822, the cyclin-dependent kinase inhibitor roscovitine, the Raf kinase inhibitor sorafenib, the Rad51 inhibitor RI-1, the PARP-1 inhibitor olaparib or the HMG-CoA reductase inhibitor lovastatin. After an incubation period of 72 h hours, cell viability was analyzed using the Alamar blue assay. Listed are the resulting IC_50_ and IC_80_ from 2–3 three independent experiments, each performed in quadruplicate.

Taken together the data show that different molecular mechanims are involved in acquired resistance of different types of UC cells to CisPt. Apparently, molecularly different sets of CisPt defence programs can become activated in individual UC cells. We hypothesize that acquired CisPt resistance in (epithelial-like) RT-112 cells might be preferentially related to protection from pro-apoptotic mechanisms, whereas gain of CisPt resistance in (mesenchymal-like) J-82 UC cells seems to be characterized by a lower level of CisPt formed DNA damage and attenuated DDR. Mechanisms of transport and DNA repair seem to be of minor relevance for aquired CisPt resistance of UC cells. Hence, therapeutic targeting of apoptosis- and/or DDR-related mechanisms are suggested as preferential to overcome acquired CisPt resistance in UC. Importantly, inhibitors of Chk might be useful to handle CisPt resistance in UC cells. Forthcoming *in vivo* studies are required to scrutinize the potency of Chk1 specific inhibitors to work against the non-responsiveness of urothelial carcinoma cells to CisPt-based anticancer therapy in a clincally relevant setting.

## MATERIALS AND METHODS

### Materials

RT-112 and J-82 urothelial carcinoma cells originate from the German Collection of Microorganisms and Cell Culture (DSMZ) (Braunschweig, Germany). Cisplatin was obtained from the pharmaceutical department of the University Hospital Düsseldorf and originates from TEVA (Ulm, Germany). The following antibodies were used: antibodies detecting Ser139 phosphorylated histone H2AX (γH2AX), H2AX (Millipore (Billerica, MA, USA)), β-actin (Santa Cruz Biotechnology (Santa Cruz, CA, USA)), 53BP1, p-p53, p-Chk1, p-p38 (Cell Signaling (Denvers, MA, USA)), p-Chk2 (Abcam (Cambridge, UK)), p-RPA32 and p-KAP1 (Bethyl Laboratories (Montgomery, AL, USA)). The antibody detecting GpG intrastrand crosslinks induced by CisPt was generously provided by J. Thomale (Essen, Germany) and has been described before [30]. The fluorescent antibodies Alexa Flour 488 and 546 were obtained from Life Technologies (Carlsbad, CA, USA). Horseradish peroxidase-conjugated secondary antibodies were purchased from Rockland (Gilbertsville, PA, USA). ATM/ATR inhibitor VE-822 (CatNo:S7102) and Wee1 kinase inhibitor MK-1775 (CatNo: S1525) were obtained from Selleckchem (Munich, Germany), lovastatin (CatNo: M2147), cyclin-dependent kinase inhibitor roscovitine (CatNo: R7772) and the pan (i.e. Chk1 and Chk2) checkpoint kinase (Chk) inhibitor AZD-7762 (CatNo: SMLO350) from Sigma Aldrich Life Science (Darmstadt, Germany), Rad51 inhibitor RI-1 (CatNo: 553514) from Calbiochem (San Diego, CA, United States), Chk1-specific inhibitor LY2603618 (CatNo: A8638) and PARP-1 inhibitor olaparib (CatNo: A4154) are from Apexbio (Houston, TX, USA) and the Raf kinase inhibitor sorafenib was obtained from Santa Cruz Biotechnology, Inc. (Heidelberg, Germany) (CatNo: Sc-220125).

### Cell culture

RT-112 and J-82 cells were grown in DMEM (Sigma (Steinheim, Germany)) containing 10% of fetal calf serum (FCS) (PAA Labratories (Cölbe, Germany) and 1% penicillin/streptomycin (Sigma (Steinheim, Germany)) at 37°C in an atmosphere containing 5% CO_2_. If not stated otherwise, treatments of logarithmically growing cells were performed 24 h after seeding.

### Determination of cell viability

Cell viability was determined using the Alamar blue assay [45]. In this assay, viable cells are detectable by their ability to effectively metabolize the non-fluorescent dye resazurin (Sigma, Steinheim (Germany)) to fluorescent resorufin. Cells were incubated for 1.5 h with the resazurin solution (final concentration 40 μM) before fluorescence was measured (excitation: 535 nm, emission: 590 nm, 5 flashes, integration time: 20 μs). Relative viability in the untreated controls was set to 100%. In addition, cell viability was also determined by use of the Neutral red assay [46]. In this assay, viable cells accumulate the red dye 2-methyl-3-amino-7-dimethylaminophenazine in lysosomes, whereas dead cells are unable to do so. Thus, the staining intensity is directly proportional to the number of viable cells. For this assay, cells were incubated for 1.5 h with the neutral red solution (Sigma Aldrich (Steinheim, Germany)) (final concentration 0.1 mg/ml) before fixation with 1% formaldehyde. Afterwards the dye was extracted with 50% ethanol and absorption was measured at 550 nm. Relative viability in the untreated controls was set to 100%.

### Flow cytometry-based analysis of cell cycle distribution and cell death

Cell cycle distribution was analyzed by flow cytometry. Adherent cells were trypsinized and combined with the medium that contains floating cells. After centrifugation (800 × g, 5 min, RT), cell pellet was washed and resuspended in PBS. Afterwards, the cells were fixed with ice-cold ethanol (−20°C, ≥ 20 min). After centrifugation (800 × g, 5 min, 4°C) the supernatant was discarded. The cells were resuspended in PBS containing RNase A (Serva Electrophoresis GmbH (Heidelberg, Germany)) (1 μg/μl) and incubated for 1 h at RT. After adding of propidium iodide (Sigma (Steinheim, Germany)) cells were subjected to flow cytometric analysis (Becton Dickinson (Heidelberg, Germany)). The SubG1 fraction was considered as a measure of dead (apoptotic) cells.

### Analysis of DNA damage induction

The formation of DNA double-strand breaks (DSBs) was investigated by measuring the level of S139 phosphorylated H2AX (γH2AX), which is a surrogate marker of DNA damage [47, 48], by Western blot analysis or by immunocytochemistry-based detection of nuclear γH2AX and 53BP1 foci. For immunocytochemical analysis, the cells were seeded onto coverslips. After treatment the cells were fixed with 4% formaldehyde in phosphate buffered saline (PBS) (MERCK (Darmstadt, Germany)) (15 min, RT) and incubated with ice-cold methanol (over night, −20°C). After blocking (1.5 h, RT; blocking solution: 5% BSA in PBS/0.3% Triton X-100, incubation with γH2AX antibody (mouse) and 53BP1 antibody (rabbit) was performed (1:500, over night, 4°C), followed by further washing with PBS/0.3% Triton X-100 and addition of the secondary fluorescence-labelled antibody (1:500, 1 h, RT, in the dark). After washing, the cells were mounted in Vectashield (Vector Laboratories (Burlingame, CA, USA)) containing DAPI. NuclearγH2AX and 53BP1 foci were counted by microscopical analysis using an Olympus BX43 fluorescence microscope and the number of co-localized γH2AX and 53BP1 foci was calculated.

The level of Pt-(GpG) DNA intrastrand crosslinks was monitored by Southwestern blot analysis. To this end, genomic DNA was isolated using the “DNeasy Blood and Tissue” kit (Qiagen (Hilden, Germany)). The concentration and purity of the DNA was measured photometrically (NanoVue^™^Plus (GE Healthcare, UK)). 0.5 μg of the DNA was diluted in 100 μl of TE buffer, denatured by heating (10 min, 95°C) and cooled on ice. Afterwards, 100 μl ice-cold ammonium acetate (2 M) was added. A nitrocellulose membrane was soaked in 1 M ammonium acetate and fixed into a slot-blot apparatus (Roth (Karlsruhe, Germany)). The DNA was transferred onto the membrane by use of a vacuum pump. After washing with 1 M ammonium acetate and water, the membrane was incubated with 5 × SSC (10 × SSC: 1.5 M NaCl, 150 mM sodium citrate, pH 7.0) for 5 min and baked for 2 h at 80°C before it was blocked in 5% non-fat milk in TBS/0.1% Tween 20 over night at 4°C. Incubation with the primary antibody directed against Pt-(GpG) intrastrand crosslinks (1:200) [49] was conducted for 1 h at RT, followed by incubation with peroxidase-conjugated anti-rat IgG secondary antibody (1:2000, 2 h, RT). Visualization of the Pt-(GpG) intrastrand crosslinks was done by chemiluminescence and autoradiographies were densitometrically analyzed. Additionally, the membrane was stained with methylene blue (MP Biomedicals (Santa Ana, CA, USA)) to ensure equal DNA loading.

### Western blot analysis

The activation status of the DNA damage response (DDR) machinery was investigated by Western blot analysis employing a set of phospho-specific antibodies, which detect prototypical factors that become activated by phosphorylation in the course of the DDR. Total cell extracts were obtained by lysing an equal number of cells in Roti^®^-Load buffer (Carl Roth GmbH (Karlsruhe, Germany)) (5 min, RT). After sonication (EpiShear™ Probe sonicator, Active Motif (La Hulpe, Belgium)) proteins were denatured by heating (5 min, 95°C) and separated by SDS-PAGE (12.5% gel). Subsequently, proteins were transferred onto a nitrocellulose membrane (GE Healthcare (Little Chalfont, UK)) via the Protean Mini Cell System (BioRad (München, Germany)). After blocking in 5% non-fat milk in TBS/0.1% Tween 20 (MERCK (Darmstadt, Germany)) (2 h, RT), the membrane was incubated with the corresponding primary antibody (1:1000, over night, 4°C). After washing with TBS/0.1% Tween 20 the secondary (peroxidase-conjugated) antibody was added (1:2000, 2 h, RT). For visualization of the bound antibodies the Fusion FX7 imaging system (PeqLab (Erlangen, Germany)) was used.

### Quantitative real-time PCR-based mRNA expression analyses

Putative markers of CisPt susceptibility were selected on the basis of a recent review by Galluzzi et al. [17] who has classified putative CisPt resistance factors of tumor cells into mechanisms of pre-, on-, post- and off-target. Based on this report we assembled a 96 well-based quantitative real-time (qRT) PCR array to analyze the mRNA expression of these factors in RT-112 und J-82 cells. In addition, mRNA expression of the epithelial marker *E-cadherin* as well as the mesenchymal marker *vimentin* and the proliferation factors *c-Myc* and *cyclinD1* was analyzed by qRT-PCR. Total RNA was purified using the RNeasy Mini Kit (Qiagen (Hilden, Germany)). The reverse transcriptase (RT) reaction was performed by use of the OmniScript Kit (Qiagen) with 2000 ng of mRNA. For each PCR reaction 40 ng of cDNA and 0.25 μM of the corresponding primers (Eurofins MWG Synthesis GmbH (Ebersberg, Germany)) were used. Quantitative real-time PCR analysis was performed in triplicates employing the QPCR-SYBR Green Fluorescein Mix (Thermo Fisher Scientific (Dreieich, Germany)) and a CFX96 Real-Time System (BioRad (Munich, Germany)) with the Bio-Rad CFX Manager 3.1 software. PCR runs (35–40 cycles) were done as follows: 95°C – 10 min; 95°C – 15 s; 60°C – 30 s; 72°C – 40 s; 72°C – 10 min. At the end of the runs, melting curves were analyzed to ensure the specificity of the amplification reaction. mRNA levels of *β-actin, GAPDH, PPIA, RPL32 and 18S* were taken for normalization. If not stated otherwise, relative mRNA expression of untreated control cells was set to 1.0.

### Statistical analysis

For statistical analysis the unpaired two-tailed Student's *t*-test was applied using the GraphPad Prism 5.01 software. *p*-Values ≤ 0.05 were considered as significant and were marked with an asterisk.

## HIGHLIGHTS

Expression of CisPt specific resistance factors differs between urothelial carcinoma cells lines

Selection of CisPt resistant UC cell variants promotes an EMT-like phenotype

Aquired CisPt resistance of epithelial-like RT-112 UC cells is related to a lower frequency of apoptosis

CisPt resistant mesenchymal-like J-82 UC cells are characterized by reduced formation of DNA damage and attenuated DDR

Acquired CisPt resistance is reversible by pharmacological inhibition of Chk1.

## SUPPLEMENTARY FIGURE



## Abbreviations

53BP1: 53 binding protein 1
ApG: adenine-guanine
ATM: ataxia telangiectasia mutated
ATP7A: copper-extruding P-type ATPase
ATR: ataxia telangiectasia mutated and Rad3-related
BCL2: B-cell/lymphoma 2
BIRC5: Survivin
BRCA: breast cancer
Chk: checkpoint kinase
CisPt: cisplatin
DDR: DNA damage response
DSBs: DNA double-strand breaks
DYRK1VB: dual specificity tyrosine-phosphorylation-regulated kinase 1B
ERBB2: oncogenic EGFR-like receptor
Ercc1: excision repair cross complementing gene 1
EMT: epithelial mesenchymal transition
GpG: guanine-guanine
GPX1: glutathione peroxidase 1
GSTM1: glutathion S-transferase type M1
HMOX1: heme oxygenase type 1
H2AX: histone H2AX
γH2AX: S139 phosphorylated H2AX
HSPA1B: heat shock protein 1B
IR: ionizing radiation
Kap1: KRAB-associated protein 1
MSH2: mutS homolog 2
MT1A: metallothionein 1A
NER: nucleotide excision repair
RT: reverse transcriptase
TC-NER: transcription-coupled NER
PARP-1: poly (ADP-ribose) polymerase 1
RPA32: replication protein A2
UC: urothelial carcinoma
UC: urothelial carcinoma
VDAC: voltage-dependent anion channel
Wee1: nuclear protein tyrosine kinase regulating G2 checkpoint
XAF1: Xiap-associated factor 1
XRCC3: X-ray repair cross-complementing gene 3.
