# Supplementary material for: Distinct mechanisms contribute to acquired cisplatin resistance of urothelial carcinoma cells
Source: Oncotarget. 2016 May 12;7(27):41320–35. doi: 10.18632/oncotarget.9321 (PMC5173062; doi:10.18632/oncotarget.9321)
Supplement: Supplementary file 1 [file oncotarget-07-41320-s001.pdf]

Distinct mechanisms contribute to acquired cisplatin resistance of urothelial carcinoma cells

Supplementary Materials

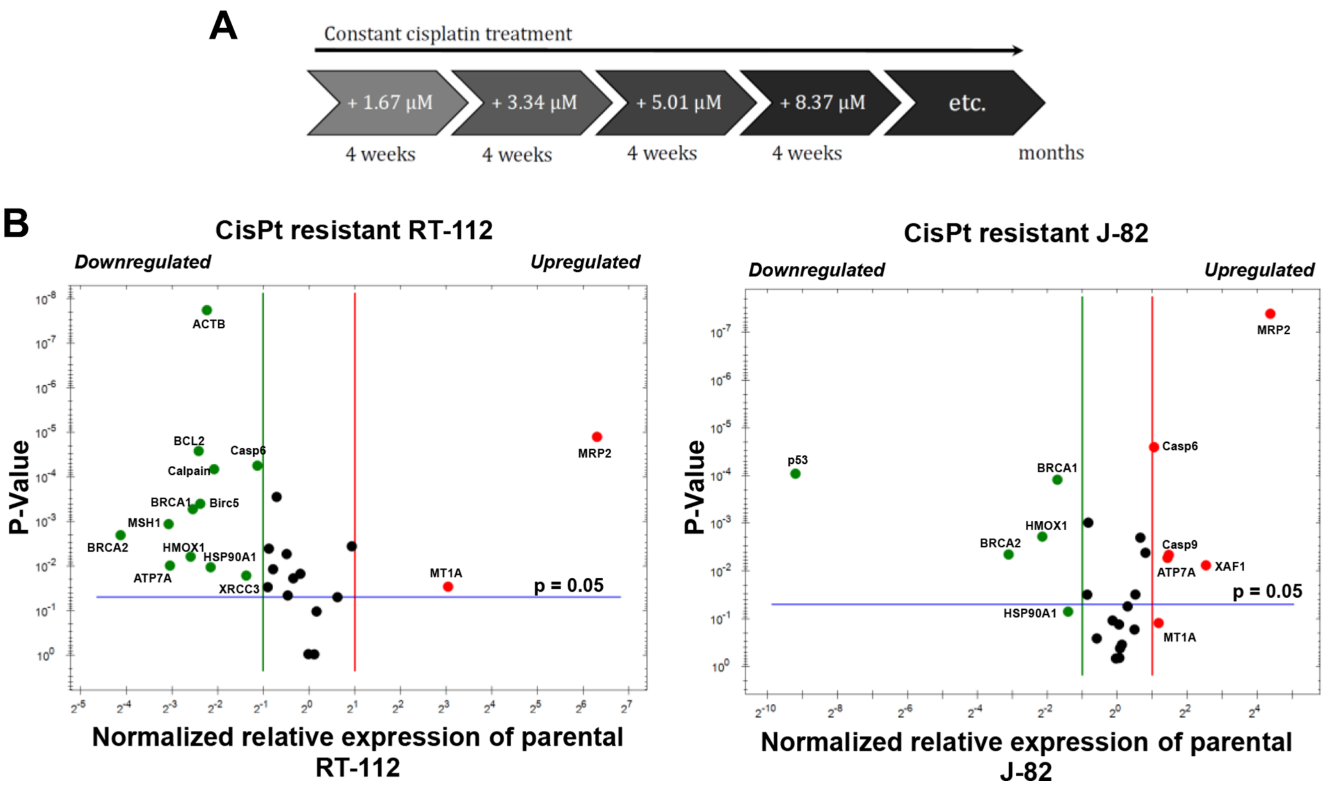

Supplementary Figure S1: Alterations in gene expression going along with continuous long-time CisPt selection of epithelial- and mesenchymal-like UC cells. (**A**) Schematic representation of the long-term CisPt selection scheme applied to RT-112 and J-82 cells. After each passage of the cells, CisPt was added at the concentration indicated and left on the cells until next passage (7–10 days later). After a total of four weeks the CisPt concentration was increased as shown. Using this selection procedure cell variants were obtained that revealed an about 3-fold and 4-fold increased CisPt resistance (as concluded from the IC_{50} values) for J-82^{R} and RT-112^{R} cells, respectively, as compared to corresponding parental cells. Comparative mRNA expression analyses shown under B and C were performed after a CisPt selection period of 4–6 month. (**B**, **C**) The mRNA expression of selected subset of CisPt-related susceptibility factors [5] was analyzed by quantitative RT-PCR as described in Methods. Relative mRNA expression in parental J-82 cells was set to 1.0. Only alterations in gene expression of ≤ 0.5 or ≥ 2.0 between parental cells (J-82) and the CisPt resistant variant (J-82^{R}) were considered as biologically relevant. Shown are the genes that are either up- or downregulated in RT-112R as compared to the RT-112 control (A) or J-82^{R} cells as compared to the corresponding J-82 parental cells (B).
